# Rethinking human resources and capacity building needs for malaria control and elimination in Africa

**DOI:** 10.1371/journal.pgph.0000210

**Published:** 2022-05-09

**Authors:** Halima Mwenesi, Charles Mbogo, Núria Casamitjana, Marcia C. Castro, Maurice A. Itoe, Friday Okonofua, Marcel Tanner

**Affiliations:** 1 Consultant, Nairobi, Kenya; 2 Kenya Medical Research Institute (KEMRI)–Wellcome Trust Research Program, Nairobi, Kenya; 3 Center for Geographic Medicine Research, Coast (CGMR(C), Kenya Medical Research Institute (KEMRI), Kilifi, Kenya; 4 Barcelona Institute for Global Health (ISGlobal), Hospital Clinic–University of Barcelona, Barcelona, Spain; 5 Department of Global Health and Population, Harvard T.H. Chan School of Public Health, Boston, Massachusetts, United States of America; 6 Department of Immunology and Infectious Diseases, Harvard T.H. Chan School of Public Health, Boston, Massachusetts, United States of America; 7 Department of Obstetrics and Gynaecology, School of Medicine, University of Benin, Benin City, Nigeria; 8 Department of Medical Parasitology and Infection Biology, Swiss Tropical and Public Health Institute, Basel, Switzerland; 9 University of Basel, Basel, Switzerland; University of Bergen: Universitetet i Bergen, NORWAY

## Abstract

Despite considerable success in controlling malaria worldwide, progress toward achieving malaria elimination has largely stalled. In particular, strategies to overcome roadblocks in malaria control and elimination in Africa are critical to achieving worldwide malaria elimination goals—this continent carries 94% of the global malaria case burden. To identify key areas for targeted efforts, we combined a comprehensive review of current literature with direct feedback gathered from frontline malaria workers, leaders, and scholars from Africa. Our analysis identified deficiencies in human resources, training, and capacity building at all levels, from research and development to community involvement. Addressing these needs will require active and coordinated engagement of stakeholders as well as implementation of effective strategies, with malaria-endemic countries owning the relevant processes. This paper reports those valuable identified needs and their concomitant opportunities to accelerate progress toward the goals of the World Health Organization’s Global Technical Strategy for Malaria 2016–2030. Ultimately, we underscore the critical need to re-think current approaches and expand concerted efforts toward increasing relevant human resources for health and capacity building at all levels if we are to develop the relevant competencies necessary to maintain current gains while accelerating momentum toward malaria control and elimination.

## Introduction

Current malaria statistics indicate that progress toward achieving malaria elimination by 2030 has largely stalled. From 2015 to 2019, cases of malaria declined only by 3% and deaths by 18% worldwide [1]. The 2020 World Malaria Report [2] concludes that the World Health Organization (WHO) Global Technical Strategy for Malaria 2016–2030 (GTS) [3] milestones of 40% reduction in malaria morbidity and mortality by 2020 will not be achieved. Countries continue to face the challenge of suboptimal uptake and scaling up of high-impact interventions to achieve high coverage and interrupt malaria transmission and infection. These interventions include testing, treating, and tracking; chemoprevention including intermittent preventive treatment in pregnancy (IPTp), intermittent preventive treatment in infants (IPTi), and seasonal malaria chemoprevention; and use of long-lasting insecticide nets, indoor residual spraying, and environmental actions such as larviciding where feasible. In addition, proper coverage in hard-to-reach areas and populations remains a challenge.

Efforts continue to try to understand the root causes of the stall and to seek solutions to roadblocks for malaria control and elimination. Recent examples include a report of the WHO Strategic Advisory Group on Malaria Eradication [4], Lancet Commission on Malaria Eradication [5], Malaria Eradication Research Agenda (malERA) Refresh series [6, 7], and WHO guidance to countries on responding to malaria in the context of the COVID-19 pandemic [8–10]. These examples illustrate an urgency to rethink efforts to control and eliminate malaria toward attaining GTS goals and milestones.

Ten of the 11 countries with the highest malaria burden are in Africa, and in 2019 the continent had an estimated 215 million cases, approximately 94% of all cases worldwide. One critical domain in the fight against malaria is Human Resources for Health (HRH) and the capacity to implement the GTS elimination agenda. Empirical evidence [2, 6, 11, 12] suggests that malaria persistence in Africa may be attributed largely to a chronic shortage and maldistribution of the existing malaria workforce, as well as a general lack of required skills and competencies for personnel engaged in decision-making, education, research, and implementation of malaria interventions. This problem calls for not only increasing the current number of workers, but also equipping the workforce with relevant knowledge and training that will help maintain current gains while accelerating momentum toward malaria elimination.

Capacity strengthening is required in all relevant areas of malaria research and development, clinical and public health provision, leadership and program management, analytical and problem-solving skills, and community engagement [2, 11, 13]—but especially in deliberate “mainstreaming” of data sciences and literacy in the training and practice of health workers at all levels to enable them to identify, evaluate, and use reliable data for decision-making. This will necessitate not only a change in training approaches at all levels but also a mindset change among all stakeholders, especially policymakers, planners, National Malaria Programs (NMPs), donors, and development partners. Considerations for the workforce must examine the “education, recruitment, employment, performance optimization, and retention” policies in each country [14]. Addressing HRH for malaria must be prioritized, despite other pressing constraints of already severely challenged health systems in many countries in Africa. Anchoring the effort on the need to achieve United Nations Sustainable Development Goals and a strong primary health care platform for accelerating progress toward universal health coverage (UHC) will expedite the process.

This paper discusses the status of the malaria workforce in terms of adequacy and skills/competencies, as well as its ability to meet GTS goals for malaria control and elimination in Africa by 2030. We conducted an extensive literature review and supplemented this with information from informal feedback with frontline malaria workers, leaders, and scholars from Africa as part of the "Rethinking Malaria in the Context of COVID-19" global engagement. Together, these data and insights highlight three main issues: 1) gaps in training needs (access, quality, and quantity) at national, subnational, and community levels; 2) inadequacy of existing technical and non-technical competencies and skills; and 3) state of available infrastructure, financial resources, and equipment. Recommendations on logistics and approaches to mitigate training/skills/competency gaps and numbers of malaria health workers, as well as making a case for creating an enabling environment with adequate resources to enable more effective implementation of impactful interventions are made.

## Challenges for human resources for health: Workforce and capacity building

A strong HRH platform in terms of the workforce and their skills/competencies in a health system is the backbone of not only better health outcomes for all but also achievement of the global Sustainable Development Goals, UHC goals, and, by extension, GTS targets. The 2010 WHO Global Policy on Recommendations on Increasing Access to Health Workers in Remote Rural Areas through Improved Retention and the 2016 WHO Human Resources for Health Action Framework [15, 16] include elements designed to address key HRH challenges including workforce shortages, misdistribution of personnel, gaps in skills and competencies, low retention, and poor motivation.

The COVID-19 pandemic not only emphasized the critical role of HRH in health systems but also amplified the serious need for skilled manpower at all levels and particularly in nursing and midwifery. Similarly, the pandemic further revealed the need for countries to recommit to and invest adequate resources in all areas of HRH [17]. The importance of this topic prompted WHO to declare 2021 the year of health and care workers globally [18].

Stalling of GTS targets over the last five years amplifies the need to rethink HRH and capacity building for malaria. According to the GTS, at least 10 countries were expected to be malaria-free by 2020, 25 countries by 2025, and 35 countries by 2030 [3]. While some progress is evident at the global level, there has been a generally poor response at regional and national levels for various reasons, including limited availability of new vector control tools; critical financial, human, and infrastructural resource deficiencies; as well as a focus mainly on biomedical skills training, might require rethinking. As in general health, effective and sustainable malaria elimination can be achievable only with enough and adequately trained human resources, an enabling infrastructure, and a functional health system. The WHO Human Resources for Health Action Framework and the recently developed WHO-sponsored Checklist for Implementing Rural Pathways to Train, Develop and Support Health Workers in Low and Middle-Income Countries are good resources to assist countries and stakeholders not only address malaria-specific HRH issues and a focus on rural, hard-to-reach areas, but also inform the needed integrated approaches to address broader areas of health in the context of limited resources [19].

### Training for the malaria workforce: A brief description

Historically, African countries have trained their health workforce and strengthened research capacity through their tertiary education and research institutions and in partnership with the WHO [20, 21] and northern development partners and training institutions. These efforts focus on training individuals in different disciplines relevant to malaria through various formats, including traditional classroom/pedagogical methods for postgraduate and undergraduate degrees and more recent eHealth/mHealth learning at tertiary and middle-level medical training colleges for pre-service and in-service diploma/certificate programs. In these contexts, training takes at least three and upwards of 12 years, depending on the discipline and degree/diploma/certification being pursued.

Continuing education and on-the-job training remain mandatory for some disciplines. Such training may include short courses (certified or non-certified) relevant to an individual’s role. Other capacity strengthening approaches include: i) internships and continuous on-the-job coaching and mentoring; ii) use of short-term consultants or long-term technical advisors, attached to NMPs for time-limited periods, to transfer specific skills through targeted malaria technical assistance on areas of need at national and/or subnational levels; iii) cross-country benchmarking exchange visits for malaria experts to learn from each other; and iv) virtual or in-person conferences to strengthen global knowledge exchange. Community of practice face-to-face or virtual platforms also have been used to strengthen capacity. Some of these approaches further allow for hands-on learning [22]. Generally, training has tended to occur away from workstations; however, creating substantial “absenteeism,” disrupting service delivery, and increasing cost of training [23].

Training for community-based health workers [24] who help bridge the gap in adequate numbers of professional healthcare workers and cater to remote underserved populations includes classroom and in some instances training in the “open air” under trees. This form of training cascades from the highest to the lowest levels of a health system. While the specifics of cascaded training may differ with the setting, the training generally starts with central training-of-trainers workshops, followed by subnational training of public health professionals and frontline providers at the health facility level who then train community health workers/volunteers (CHWs) at the community level. Training for the different levels takes several days depending on the subject and abilities of instructors and learners.

The training that CHWs receive is recognized by formal health services, yet their certification or accreditation, if it occurs, is not part of the higher education certification process—which is key to recognition and professional career development and promotion at all levels. Also, continuous education, resources, and self-development opportunities vary for this cadre of frontline workers. Thus, CHWs, although perceived as an essential cadre, yet have not been fully utilized in Africa, often due to lack of resources and adequate planning [24].

These methods and approaches have worked relatively well for several decades, enabling countries to respond to global, regional, and national agendas and malaria control and elimination targets. However, the increase in malaria burden, population growth, biological threats (e.g., insecticide and drug resistance), the need for equity, and mounting pressures on health systems from other communicable and non-communicable diseases are challenges related to capacity building and increasing workforce size that must be addressed to achieve 2030 targets and beyond.

### Specific malaria capacity expert base and workforce bottlenecks

Most bottlenecks outlined below are policy-related but actionable. They are informed by challenges identified across several malaria technical and service delivery areas as well as by stagnation of various elements in the fight against malaria.

#### Training, recruitment, and retention inadequacies

Malaria is a complex infection and disease. Its epidemiology is affected by many factors inherent in the disease and its transmission, as well as by social determinants. Therefore, the malaria response requires continuous research and development as well as a review of tools and approaches, which necessitates training and retraining of the requisite workforce. Such efforts must occur in parallel with continued implementation of ongoing interventions, especially in countries progressing from control to elimination. The high cost and time lags in advanced training of scientists and researchers who form the malaria expert base not only affect the pipeline of available experts but also negatively impact timely translation of research evidence into practice [25, 26]. This is compounded by the fact that new knowledge often has to be synthesized at the global level for standard normative guidance, trickling down to the countries where it must be adopted and adapted to different socioeconomic and environmental contexts and health system levels. Unfortunately, dissemination of new global knowledge and updates from national to subnational levels and service delivery points where interventions are implemented is not always optimal.

This review noted a concerning imbalance in the focus of training (Table 1) that has favored basic and biomedical sciences while neglecting knowledge generation and the critical need for a workforce with skills in operational/translational/implementation sciences [2, 27]. For example, articles and consultative meeting reports typically indicate that there are insufficient numbers of entomologists, genomic experts, and data scientists critical for surveillance, monitoring and evaluation, modelling, and logistics for supply chain management [2–4, 6, 11, 28, 29]. However, the dismal number of translational/implementation scientists across the board, especially in social sciences (including sociologists, anthropologists, behavioral scientists, specialists in advocacy and health diplomacy, health promotion and communication experts, policy analysts, health economists, resource mobilizers, gender and human rights specialists, program/project managers, team leaders, and community-based health systems specialists), also is acknowledged as a critical bottleneck but does not receive the same impetus as biomedical sciences to reverse the situation.

**Table 1:** Capacity strengthening and training for malaria: Current status.

Current Status	Core Courses	Specialized Courses	Gaps/Weaknesses	Opportunities	Threats
Biomedical Sciences	Epidemiology	Surveillance and stratification	Micro-stratification Medical entomology Lack of good data sciences Limited number of pharma scientists	A plethora of existing materials from WHO, PMI/CDC, Global Health Network, EDCTP, Harvard-ISG-Swiss TPH consortium, and Networks in Asia and ACTMalaria Existence of a substantial mass of African centers of excellence for malaria research and teaching in Central, East, Southern and West Africa that can address the identified weaknesses	Lack of coordination and common training strategy Lack of real estimates of need, and therefore failure of implementing effective strategies Territorialism Lack of funding and lack of interest in working in an area that might become obsolete when malaria is eradicated Lack of political commitment and country ownership Over-reliance of countries on external funding Perceived dominance of the malaria response by the North Data illiteracy at all levels of the health workforce and in all sciences (biomedical and social) Huge challenge to regulate and reach the large number of this cadre especially in urban areas.
	Entomology and vector control	Vector resistance and surveillance			
	Diagnostics and case management Pharmaceutical Sciences	Microscopy, Therapeutic Efficacy Studies, drug resistance Chemoprevention Drug discovery, Dispensing, pharmacovigilance, etc.			
Implementation and Operational Sciences	Planning and management of malaria programs	Leadership training, advocacy and social mobilization	Health information sciences Logistics and supply chain management Policy dialogue, analysis, and development	Public health schools could collaborate with departments of humanities to provide degree, certificate, and short-term courses to address identified gaps Focus on training mid-level career health workers Training of CHWs could include training of informal drug dispensers on whom many communities depend for first treatment of perceived malaria symptoms	
		Resource mobilization	Operational research Community engagement Training in ethics Human rights and gender Health economics Multi/trans/intra disciplinary approaches Analytical problem-solving skills Partner coordination		

Also lacking is global agreement on a malaria training strategy and curricula aligned to current WHO global strategies and specific country malaria control/elimination needs. The need to address this gap resulted in the proliferation of many well-meaning organizations and institutions all working individually, without coordination around an overall set of training aims that are monitored over time. Thus, training approaches are fragmented and often even disconnected from national and subnational strategies. This raises important questions not only about the quality of courses and training but also whether they have sufficiently clear goals and objectives to address real-world gaps. Further, at all levels of training, adequate supportive supervision and post-training follow-up to reinforce learning and update the knowledge base, especially for frontline and community-based health workers, is vital yet lacking. In addition, when supervisory activities or visits take place, identified issues are not always addressed. Lack of resources and/or adequate opportunities to apply knowledge and skills learned after training also is a key issue.

Nevertheless, there is a sizable well-trained base of health experts in Africa capable and committed to integrated malaria control and elimination that has contributed to the progress achieved to date. While these experts were trained locally and globally, there is a general lack of follow-up and measurement of the impact of advanced/specialized training on the malaria response, and especially on the capacity of home institutions to provide the right environment and support for globally trained experts to further develop their capacities once back in their home countries/institutions. As reported by Woyessa et al. [12] and Juma et al. [30] and echoed in the feedback of frontline malaria workers for this review, other post-training constraints abound for individuals trained in malaria-relevant skills and competencies at all levels, from scientists to CHWs. There is a lack of career pathways and personal/professional growth, which is further compounded by poor remuneration and lack of incentives. This leads to high staff turnover and brain-drain, necessitating costly refresher and continuous trainings. Lack of proper planning and management of transfers and retirements also negatively impacts the health workforce [14]. In addition, maldistribution or inequitable distribution of the health workforce as well as political appointments of NMP personnel undermine proper workforce deployment and negatively impact the effectiveness of malaria control/elimination programs. “Siloed” training without integrated approaches that have the potential to not only expand and optimize the malaria expert base but also that for other vector-borne and infectious diseases represents another missed opportunity.

Our review also revealed a lack of information (database or registry) on HRH specifically for malaria in Africa. While this also has been reported for tuberculosis [31], lack of clarity on the current size and competencies/skill sets of a health workforce can prevent appropriate short- and long-term planning, including adequate investment in pre-service training at all levels and strategic recruitment, replacement, and deployment. This type of information also is critical for forecasting future competencies and skills needs as well as other important matters such as financial planning [14, 32].

#### Weak multisectoral coordination and collaboration

Although frameworks to address malaria through a socioeconomic development lens via multisectoral, intersectoral, and across inter- and intra-national boundaries approaches have long existed [33, 34], their implementation and results are not apparent. The frameworks have addressed various aspects of multisectoral actions between the health sector and non-health partners in finance, public services, agriculture, education, water, sanitation/hygiene, defense/security, transportation, public works/housing/urban planning, and the private sector. However, collaboration across these sectors generally remains weak, especially in terms of education and training at all levels, programming, and workforce management.

This lack of collaboration undermines maximization of potentially available human and financial resources that could be leveraged and rallied around malaria responses where most needed. Further, cross-training, especially for NMP managers with personnel from the other sectors, might open non-traditional platforms to facilitate better workforce management as well as deeper penetration and access to health for remote hard-to-reach populations and geographies. Realization of the importance of this aspect of the malaria response has stimulated re-thinking of non-health sectors that must be included in the fight against malaria, including extractive industries, humanitarian emergency response, primary education, and tourism, as well as better elucidation of what multisectoral action on malaria should look like [35]. However, training and joint programming are not included in the four broad categories proposed for cooperation on malaria control.

#### Universal health coverage, community engagement, and gender mainstreaming issues

The UHC initiative, which should be anchored on a strong primary health care platform to enable realization of Africa Union’s targets for 2030 and beyond [36], has experienced slow adoption to date. A recent report on the Status of Universal Health Coverage in Africa: Report of the Africa Health Agenda International Conference Commission [37] indicates that African countries are still struggling to create proper roadmaps to reach UHC targets, exhibiting low achievement in almost all key indicators including the priority of an increased, skilled, and competent health workforce, especially in public health skills. This also may partly account for the observed stalling in meeting 2030 GTS targets.

With regard to community engagement, home-grown solutions grounded in local knowledge and local actors generally are more sustainable compared to externally driven solutions. However, although there have been commendable efforts to engage communities and to institutionalize and mainstream CHWs into formal health systems, success in different countries is variable. This appears to be mainly due to a somewhat narrow focus on a three-decade-old definition of who a CHW is—“community health workers should be members of the communities where they work, should be selected by the communities, should be answerable to the communities for their activities, should be supported by the health system but not necessarily as part of its organization, and have shorter training than professional workers.” [38]. This definition may need to be adjusted to accommodate a more inclusive people and household-centered approach, which would facilitate exploration of other possible models described in the next section and also reconsider “accountability” arrangements.

Further, evidence indicates that 70% of the global health workforce is female, especially at the frontline and community levels [39]. These workers generally have low levels of education, receive minimal training, and are under-resourced, overworked, and underpaid or unpaid. Also, there are few women in sciences in general, few in malaria leadership, and even fewer in global health leadership. The gross under-representation of males at the frontlines should be addressed, as these constraints and lack of gender-sensitive programming for malaria also may be linked to chronically low uptake of core malaria interventions.

## Opportunities for improvement and recommendations

These identified areas of training and capacity building or strengthening indicate opportunities to improve and move further toward achieving GTS milestones and goals. Some of the opportunities are already in place and just need to be reinforced; others must be assessed; and yet others are innovative and will require bold global, national, and political commitment because they have cost implications. For example, it will be necessary for countries to include ring-fenced training and capacity building in NMP budgets. The proposed recommendations also may necessitate long-term periodic policy changes, guideline updates, and dissemination due to emerging new evidence until malaria is eradicated. Our review of the literature complemented by informal feedback from frontline malaria workers has informed the following opportunities and innovative approaches for capacity building and workforce enhancement that could be scaled and/or retooled for this purpose.

### Strengthening capacity for malaria control and elimination

Malaria endemic countries’ ministries of health and education as well as academic and research institutions and other relevant sectors, working with WHO and partners, should assess the impact of training time lags on the malaria response, similar to assessments of the impact of time lags in getting research evidence into practice [26]. The merits of refocusing efforts on training mid-level and frontline health workers also should be assessed [40]. This presents an opportunity for countries to address critical elements of capacity strengthening in partnership, coordination, and collaboration with other health and non-health sectors in an integrated manner.

Investment in integrated malaria capacity-building has the potential for spillover effects on other health interventions and programs, as well as on the entire health system and society at large. Countries must spearhead and own this dialogue as they engage with partners including the Africa CDC, WHO and WHO Academy, donors, and other development partners. This would entail agreeing on a training strategy and curriculum or series of curricula for training at different levels and for different cadres of malaria workers. This would then be adapted and tailored to specific country needs, ensuring the countries own the entire process—from planning to implementation and post-training follow-up, which is critical in capacity-building/strengthening. It is likely that having a standard, agreed-upon approach would enable its coordinated delivery through multiple agencies/funders. Such standardization would also address issues with quality of courses and training as well as measurement and evaluation of the training over time. Ownership of the process by national governments could help them better plan and focus their domestic resources on training. It also would act as an accountability measure for all stakeholders to assure sustainability.

We suggest some pathways to curriculum/certification standardization to consider. There already exists in the malaria space, the diagnosis through microscopy certification process that could be a pathfinder for other skill sets in malaria. Also, the malaria community could borrow a leaf from the Global Health Network, a platform that runs a professional competence scheme for clinical trials in partnership with the UNDP/UNICEF/WHO Special Program for Research and Training in Tropical Diseases (TDR) which utilizes the power of high-quality resources, virtual learning and a standardized WHO approved curriculum. The training allows an individual progress through various levels—from the most basic to the expert [41]. We envision a similar scheme for malaria control and elimination, which would be coupled with some form of agreed upon of certification, or through standard accreditation processes spanning all aspects of malaria control and elimination regardless of the individual’s basic training (i.e., biomedical, public health or social sciences). It would be important that these utilize existing regional and/or in-country academic and board certifying professional organizations, and governmental resources and personnel. Africa currently has centers of excellence in malaria research in Ghana, Kenya, Malawi, Mali, Tanzania to name but a few. This critical mass of experts, together with other malaria global centers and experts could quickly get this urgent process going. Completion of the next steps of the “Informal Consultation on the Development of a Capacity Building Strategy for Malaria Control and Elimination,” convened by the WHO’s Global Malaria Programme in March 2018, where these proposals could be further interrogated, with an expanded stakeholder base (e.g., relevant non-health partners) to make it inclusive, transparent, and participatory should be expedited.

To help tailor solutions, local universities, and biomedical/public health institutions—on their own or with south–south and/or north–south partnerships—should take the lead in rethinking how to deliver targeted training, which will serve capacity building/strengthening needs at the individual, institutional, and health systems levels. South–south institutional collaborations must be prioritized and emphasized, while northern training institutions should only jointly offer malaria training together with disease-impacted southern counterpart. Additionally, NMPs will need to form new partnerships with humanities, social sciences, and data sciences departments at universities and training institutions to enlist experts in disciplines that inform operational, translational, and implementation aspects of malaria control, including social and behavior change communication and mobilization, policy analysis and development, gender and human rights, and project management. These soft sciences have the potential to improve uptake of existing tools and interventions and ensure they are fully optimized through compliance by providers and users. Thus, existing partnerships with biomedical departments should be strategically reoriented to areas of most need, such as a mechanism to facilitate faster and systematic dissemination of new global knowledge and updates at the country level, and to ensure these seamlessly cascade from national to subnational and community-level service delivery points. Funding agencies also should rethink their agendas and focus their attention on what countries need by promoting demand rather than offer-driven solutions for identified needs via research and training calls/grants.

At the NMP level, improvements that could better serve the malaria response include deliberate periodic analytical assessments of gaps in skills/capacities in each endemic country to strategically tailor short- and long-term training and/or technical assistance to quickly respond to needs. These regular technical or service delivery assessments also could include reviewing of interventions, approaches, and tools as part of ongoing training. Methods and approaches of “in-service” training that do not take malaria workers from their day-to-day jobs should be prioritized at all levels [23, 42]. This ensures that core work of the workforce is not affected by frequent/long absences from their jobs. Further, implementing partners/agencies (local and international non-governmental organizations) have capacity strengthening models that are currently project-based that should be evaluated to assess their cost-effectiveness and scalability. This includes the Long-Term Technical Assistance program [22] and coaching, mentoring, and cross-country/state/county study tours. Regular, appropriate, supportive post-training supervision is vital and must be strengthened at the NMP level to reinforce newly acquired knowledge and skills for the malaria workforce. For example, this could address a critical and perennial problem of health providers not following protocol on parasitological testing of fever cases before treatment.

Further, countries must institute strategic multisectoral, intersectoral, and cross-border collaboration in relation to training to maximize available human and financial resources that could be leveraged and rallied around the malaria response at country and regional levels. Frameworks and guidelines on how to implement multisectoral approaches exist, but they are silent on how training could be carried out within their ambit. Cross-training with personnel from neighboring countries and other relevant disease programs as well as non-health partners might open non-traditional platforms to optimize health workforce teams that can work across diseases, leveraging synergies and optimizing the available health workforce, especially those working in remote geographies. It is imperative that all stakeholders in health, including ministries of health, health professional regulatory boards, professional associations, training institutes, employers, and workers’ representatives, work together to implement successful changes in training and capacity strengthening for malaria [41]. Innovative strategies for broader gender diversity, inclusivity, mainstreaming at all levels, and meaningful engagement of the private sector in this process are highly desirable. An urgent action would be for the malaria community to also explore how large-scale conglomerate industry handles cross-sector training.

### Effective and/or innovative community engagement models

Recent evidence indicates that countries that eliminate malaria have relied on cadres of CHWs, paid workers or volunteers who detect, diagnose, and sometimes treat malaria [43]. The recent WHO deep dive into what it will take to engage communities—successfully culminating in development of the Community Engagement Framework for Quality, People-centered, and Resilient Health Services [44]—is an opportunity that could leverage the full potential of the CHW movement, which is already established in most malaria-endemic countries. We posit that it will be necessary to broaden the definition of a CHW to encompass other categories of individuals who could provide frontline health service delivery periodically in the short-term and permanently in the longer term. Another resource that could be useful in further articulating meaningful and effective community engagement is the second edition of the Clinical and Translational Science Awards Consortium Community Engagement Key Function Committee Task Force on the Principles of Community Engagement [45]. Community engagement must emphasize involving communities meaningfully in co-creation of solutions to jointly identified problems from conception to implementation through shared responsibility and with well-defined roles and responsibilities of all partners.

WHO estimates that 18 million more health workers are required to achieve UHC by 2030 in low- and lower-middle-income countries [46]. We propose that new community service delivery models that have potential to also serve hard-to-reach areas [19] can help address the chronic shortage of HRH in general, in order to increase and optimize the health workforce for malaria in particular. The WHO High Burden to High Impact and E-2025 initiatives [47, 48] present early opportunities to pilot and/or strengthen the models below.

○ An estimated 64 million youth are unemployed globally, the majority of whom are in Africa [49]. Careful selection, recruitment, training, and deployment of large numbers of unemployed youth and young adults who have requisite levels of education for specific tasks in malaria control/elimination could exponentially increase frontline health workers. The youth could be trained to perform tasks including community surveillance, case investigation, social and behavior communication/information and education communication to improve treatment-seeking behaviors, uptake and reach of seasonal malaria chemoprevention, compliant and consistent use of long lasting insecticidal nets (LLNs), community intermittent presumptive treatment of pregnant women (IPTp) and intermittent presumptive treatment of infants (IPTi), diagnosis with rapid diagnostic tests and treatment, and referrals. Their jobs could be treated as short-term seasonal work during malaria surges or epidemics, a concept that is acceptable in other areas such as agriculture. A framework already exists that could be used to assess feasibility and scalability of this proposal [50]. Some countries also have youth employment strategies that could be encouraged to incorporate malaria control activities into their plans. For example, Rwanda has an active Youth Against Malaria Organization; Kenya is using a youth employment strategy to improve urban slums, which could be tapped for malaria control/elimination; and there are likely other examples from other countries. The recent Africa Health Agenda International Conference Commission [37] report emphasizes the critical need to harness and empower African youth and women with knowledge and skills to enable them to play a more significant role in UHC delivery. Gender diversity, inclusivity, and mainstreaming must be at the core of women and youth empowerment.
○ Training a cadre of health workers who would be deployed in their local areas through collaboration between NMPs and technical/vocational education and training institutions is another possible route to enhance the malaria and broader health workforce. These institutions can and in some countries do train paraprofessional health cadres that could be further trained to supervise CHWs during “seasonal malaria surge-support” periods, increasing support and accountability at this level while increasing the health workforce [22]. El Salvador used a cadre of “epidemiology assistants” and “entomological assistants” who worked side-by-side with volunteer community or “Col Vol” health workers—but also acted as the first tier of supervision for the “Col Vol” workers with impressive results in decline of malaria in the country [51]. These “Col Vol” workers also were trained and strategically deployed according to macro- and micro-stratification needs, especially during high-malaria season periods. In February 2021, El Salvador was declared malaria free.
○ Training of high school CHWs to serve underserved communities in their localities could provide a health career pipeline as well as mentoring for underserved students and could promote health education and health literacy in schools and communities. This strategy has the potential to keep youth in school and to produce health workers for tomorrow [52]. The model has been successful in the US, and frameworks that could be adapted globally have been developed. Several countries including India, Indonesia, Tanzania, and Zambia also have implemented this strategy with success.
○ Leveraging the large numbers of undergraduate university students and government pre-service Youth Training Programs available in most malaria-endemic countries by creating rotational/internship programs to coincide with high-burden malaria seasons could be explored during which the students/trainees could deliver community malaria services under supervision. This could be linked to academic credits toward students’/trainees’ degrees/diplomas/certificates, creating a win–win situation for both students/trainees and communities. Also, many countries have unemployed graduates from all disciplines who also could be targeted for training in appropriate skill sets for short-term surge-support for malaria control and elimination.
○ While faith-based organizations and civil society organizations exist in all malaria-endemic countries, they have not been fully exploited in the fight against malaria. Further, where these organizations are active, they might not be inclusive of all stakeholders. Together with engagement of traditional leaders, partnering with such organizations where appropriate could expand the workforce base beyond clinical services and especially enhance social mobilization, behavior communication, and advocacy on malaria. This point is further elaborated under key theme #3 in the related paper in this series (“Rethinking Integrated Service Delivery for Malaria”).
○ Due to changing demographics, Africa has a large reservoir of retired university professors and medics who also could be utilized to provide training and/or advice to NMPs as required.

The above suggestions could be operationalized through one of the key areas of collaboration agreed upon in a memorandum of understanding signed between the Africa Union Commission and WHO [29], aimed at assisting the African region through the Africa Centers for Disease Control and Prevention (Africa CDC) by supporting efforts to strengthen the health workforce in Africa Union member countries. This could be considered part of the proposed establishment of the African Volunteer Health Corps and rational allocation and use of existing resources, including HRH to ensure realization of UHC goals.

### Strengthening of HRH information systems

Africa, which carries 17% of the world’s population, accounts for the highest global burden of disease at 23% [37] but has only 3% of the global health workforce [53]—making addressing HRH issues an emergency. As a matter of urgency, countries and partners should systematically assess and collect HRH information for malaria and other disease control programs for synergy and integration purposes, to enable a rapid response to resolving workforce issues such as hiring, retention, and redeployment. Countries should be encouraged to create national HRH databases/registries that include all cadres of health workers from doctors to CHWs, and NMPs should include HRH budgeted development plans in national malaria strategic plans, which would be the best platform to address workforce and training needs for malaria. The plans should critically look at issues of attrition through brain drain, retirement, career mobility, and growth as well as retention at all levels. Robust expansion of malaria interventions over the past decade has been accompanied by significant requirements for an increased workforce and expert base at national, district, and community levels. Therefore, deployment of health workers to cater to expanded interventions must be strategic and should consider new roles and structures as countries progress from control to elimination. For more on the issues of data in malaria control and elimination, please see the related paper in this series (“Rethinking Integrated Service Delivery for Malaria”).

### Strategic deployment and optimization of roles of the malaria workforce

In a short period, epidemiological/entomological stratification of malaria in countries has enabled definition of malaria risk, and resultant targeted interventions have paid dividends. Countries are better prioritizing intervention mixes and resources in strata with the highest burdens. Strategic deployment of malaria teams with skills aligned to the needs of each stratum would translate into high coverage, compliance, and impact of interventions. It is recognized that not all countries know what their needs are or have all the right skills mixes, therefore, this also acts as a call for countries to conduct needs assessments to identify their gaps. Nevertheless, deliberate and rational planning and distribution of the malaria workforce could go a long way in progressing countries along the elimination continuum. This has been demonstrated in El Salvador, where malaria risk and corresponding needs were purposely used to determine the numbers and skill sets of “Col Vol” workers selected/distributed to serve specific epidemiological strata, with great success despite the country experiencing a war situation [51].

### Incentivization of the malaria workforce

Aside from lack of skills and relevant competencies to support elimination goals, the current malaria workforce is unmotivated due to low remuneration. This phenomenon leads to health workers shifting to better-paying jobs in non-governmental organizations, the private sector, and international organizations (internally and externally) or changing careers entirely, leaving an inadequate pool of personnel to sustainably stem the attrition and thus achieve GTS elimination goals. Motivation and retention packages for malaria workers that could stymie brain-drain from NMPs while motivating personnel and increasing ownership of malaria programs may include financial (better salaries, school debt forgiveness, scholarships), educational, personal, and/or professional growth support at all levels [12]. Further, it has to be emphasized again that women and youth have a right to meaningful participation in health in general, and malaria matters in particular, yet remain significantly underrepresented, especially in leadership levels. This is not only a gender equity issue, but also an important incentive area which should be tackled through career advancement opportunities to leadership positions for women and youth.

### Political commitment and funding

The Africa Union, regional health organizations in Africa, and Africa CDC are well-placed to be flag bearers and champions for supporting calls for governments and donors to commit adequate domestic and external resources for workforce enhancement and training at all levels, as well as to push for regional and cross-border efforts to ensure GTS goals are achieved and that no one is left behind. Further, civil society organizations should be encouraged to hold governments accountable for their pronouncements of commitment to ensure these become reality, especially in relation to HRH, primary health care, UHC, expenditure for healthcare, research funding, and general strengthening of health systems. The COVID-19 pandemic illustrated the ability of African governments to act quickly and decisively [54]. African governments can likewise spearhead reinvigoration of the malaria response on the heels of the pandemic. There needs to be intentional capacity building for decision-makers through various forums convened by instruments such as the Africa Union, African Leaders Malaria Alliance, and regional health organizations. International development partners also must reconsider their relationship with malaria-endemic countries and their contribution to the current high dependency of countries on donor funding. Change will have to come from both sides.

Further, due to similar needs across vector-borne diseases, other infectious diseases, and in reproductive, maternal, neonatal, and child health, a shift and focus on integrated training is imperative. We must collectively make deliberate decisions to do things differently by urgently addressing the identified issues, reinforcing what is working and discarding what is not working.

## Conclusion

This report highlights the variation in malaria workforce availability and the gaps and need for a health workforce and its required competencies/skills for malaria control and elimination in Africa. This evidence calls for re-examining current approaches as well increasing continuous and concerted efforts toward capacity building for biomedical and social scientists, public health specialists, mid- and lower-level health cadres, and decision-makers to equip them with relevant competencies and skills that will enable them to maintain current gains while accelerating momentum toward malaria elimination. We propose stakeholders who should spearhead the rethinking/retooling of capacity building and workforce enhancement as well concrete approaches that could be quickly explored and implemented. We emphasize the need for all stakeholders to collaborate and coordinate their activities while placing the ownership of relevant processes to malaria endemic countries. This implies that any efforts to enhance the workforce and setting of standardized and tailored training and capacity building should primarily be demand-driven, as opposed to often offer-driven earlier efforts. Consequently, enhanced long-term investments to massively increase the size and skill sets of professional and frontline cadres in malaria and other vector-borne disease-endemic countries as well as for peripheral healthcare and promotion should be an absolute requirement for any strategic and operational decision embraced by international, regional, and national stakeholders in malaria control and elimination as well as the entire global health agenda.

Nevertheless, even as we advocate for a competent and skilled malaria workforce, we also caution against compartmentalized training and encourage a holistic view of the problem that calls for an integration of different control programs to maximize effect and optimize resources. The COVID-19 pandemic has revealed and amplified key issues and left in its wake significant lessons in this regard.

The literature is awash with numerous global and regional commitments to HRH, primary health care, UHC, and community engagement in the form of pronouncements, frameworks, memorandums of understanding, and strategies that if implemented could address the identified issues in a short period of time. However, if they remain aspirational and rhetorical, and without an accountability mechanism with attached sanctions to ensure all stakeholders involved in malaria control and elimination efforts play their part, the desired change will continue to be a mirage—2030 will be another missed opportunity.

## Supporting information

S1 Text "Rethinking Malaria in the context of COVID–19," a global engagement organized by Harvard University. (DOCX)
