# Supplementary material for: Rethinking human resources and capacity building needs for malaria control and elimination in Africa
Source: PLOS Glob Public Health. 2022 May 9;2(5):e0000210. doi: 10.1371/journal.pgph.0000210 (PMC10021507; doi:10.1371/journal.pgph.0000210)
Supplement: S1 Text — (DOCX) [file pgph.0000210.s001.docx]

**S1 Text: "Rethinking Malaria in the Context of COVID–19," a global engagement organized by Harvard University.**

Harvard University [Internet]. Boston: Rethinking Malaria/COVID19; c2021 [cited 2021 Sept 1]. Defeating Malaria: From the Genes to the Globe Initiative; [about 3 screens]. Available from <https://www.defeatingmalaria.harvard.edu/rethinking-malaria/>.
